# Clinical study of the posterior gastric artery and the lymph nodes around it in patients with gastric cancer

**DOI:** 10.1186/s12957-024-03373-x

**Published:** 2024-04-10

**Authors:** Kexin Wang, Haitao Duan, Maohua Wei, Liang Cao, Jian Zhang, Chi Zhang, Pin Liang

**Affiliations:** 1 Department of General Surgery, First Affiliated Hospital of Dalian Medical University, Dalian, 116011 P.R. China; 2 Chengda Hospital, Dalian, 116007 P.R. China

**Keywords:** Gastric cancer, Posterior gastric artery, Lymph node metastasis

## Abstract

**Objectives:**

This study aims to gather and analyze the anatomical characteristics of the posterior gastric artery (PGA), investigate the presence and metastasis of lymph nodes around the PGA in patients with gastric cancer. Additionally, the study aims to analyze the relationship between the PGA and its surrounding lymph nodes and the clinicopathological features of patients with gastric cancer.

**Methods:**

This study consisted of a cross-sectional analysis of data from 52 patients with gastric cancer who underwent total or proximal gastrectomy at the Department of Gastrointestinal Surgery, First Affiliated Hospital of Dalian Medical University, between January 2020 and November 2022. Intraoperative exploration was performed to determine the presence of the PGA, and patients with the PGA were assessed for relevant anatomical characteristics, including the length of the PGA and the distance from the root of the PGA to the celiac trunk. Dissection of lymph nodes around the PGA was also performed. Statistical methods were employed to describe and analyze the data regarding the presence of the PGA, as well as the presence and metastasis of the lymph nodes around the PGA. Additionally, the study identified clinicopathological factors associated with these conditions.

**Results:**

The PGA was identified in 39 (75.0%) out of 52 patients with gastric cancer, exhibiting a mean PGA length of 3.5 ± 0.8 cm and a mean distance from the root of the PGA to the celiac trunk of 6.7 ± 1.7 cm. Among the 39 patients who underwent dissection of lymph nodes around the PGA, 36 lymph nodes around the PGA were detected in 20 patients. Analysis of factors associated with the presence of lymph nodes around the PGA revealed a significant correlation with the macroscopic type of the tumor and the total number of dissected lymph nodes (*P* = 0.007 and *P* = 0.022, respectively), with a larger number of total dissected lymph nodes being an independent factor (*OR* = 1.105, *95%CI*: 1.019–1.199, *P* = 0.016). Furthermore, analysis of risk factors for metastasis of the lymph nodes around the PGA demonstrated that the total number of metastatic lymph nodes, No.3 lymph node metastasis, and No.11 lymph node metastasis were associated with metastasis of the lymph nodes around the PGA (*P* = 0.043, *P* = 0.028, and *P* = 0.020, respectively).

**Conclusion:**

The PGA exhibits a high incidence. It is essential to carefully identify the PGA during procedures involving the PGA and consider appropriate preservation or disconnection of this vessel. The presence of lymph nodes around the PGA is not an isolated occurrence. Gastric cancer can result in metastasis of the lymph nodes around the PGA. Although the overall risk of metastasis of the lymph nodes around the PGA is low in patients with gastric cancer, it increases in the presence of conditions such as No.3 lymph node metastasis, No.11 lymph node metastasis, advanced tumor stage, and extensive metastases in other regional lymph nodes.

**Supplementary Information:**

The online version contains supplementary material available at 10.1186/s12957-024-03373-x.

## Introduction

Gastric cancer is a malignant tumor that originates from the epithelial cells of the gastric mucosa, making it one of the most prevalent malignancies. According to the most recent epidemiological data from 2020 [1–3], gastric cancer ranks as the fifth most prevalent cancer worldwide and the third most prevalent in China. Additionally, the mortality rate for gastric cancer is the fourth highest globally and the third highest in China. Currently, scientific research on gastric cancer, along with its detection, prevention, and treatment modalities, continues to advance. However, there is still significant room for improvement.

The primary approach to treating gastric cancer involves comprehensive treatment, with surgery as the mainstay. Nowadays, surgery remains the cornerstone of gastric cancer treatment and plays a crucial role in achieving a cure [4]. Considering that lymphatic metastasis is the primary route for gastric cancer metastasis, surgical resection of resectable gastric cancer should be complemented by lymphadenectomy in the tumor’s drainage area to achieve a complete cure. Although standardized criteria for gastric cancer surgery are becoming well established, there are still unresolved issues that lack wide acceptance. Consequently, further basic and clinical studies are necessary to address the remaining unclear and controversial questions. This study specifically investigated the posterior gastric artery and the lymph nodes around it in patients diagnosed with gastric cancer.

The posterior gastric artery (PGA) was recognized as an official anatomical term by the International Anatomical Nomenclature Committee in 1980 [5]. Previous studies have reported a wide incidence range of the PGA, varying from 4–100% [6, 7], potentially attributed to variations in the definition of the PGA across different studies. The accepted consensus is that the PGA originates from the middle of the splenic artery or its upper pole branch, situated posterior to the gastric body. It ascends behind the peritoneum of the lesser sac, extending towards the gastric fundus, and primarily supplies blood to the upper posterior wall of the gastric body, which is in close proximity to the cardia and gastric fundus. The lymphatic distribution and drainage pattern of the stomach significantly influence the extent of lymphadenectomy in gastric cancer surgery. The Japanese Classification of Gastric Carcinoma’s criteria [8] for regional lymph nodes of the stomach specify clear regional lymph nodes around all major perigastric vessels, except for the PGA, which lacks clear definition within any regional lymph node category. Previous studies primarily focused on observing and documenting the anatomical features of the PGA, with a dearth of clinical studies. Regarding lymph nodes around the PGA, only one relevant study has been conducted to date [6].

Our study enrolled 52 patients who underwent gastric cancer surgery at the Department of Gastrointestinal Surgery, First Affiliated Hospital of Dalian Medical University, between January 2020 and November 2022, based on specific inclusion criteria. Data regarding the PGA and the lymph nodes around the PGA were collected through intraoperative and postoperative procedures. The objectives of our study were as follows: to collect and analyze the anatomical characteristics of the PGA, to clarify the presence and metastasis of the lymph nodes around the PGA in patients with gastric cancer, to investigate the relationship between the lymph nodes around the PGA and lymphatic metastasis of gastric cancer.

## Materials and methods

This study was a cross-sectional study by collecting and analyzing data of patients with gastric cancer who underwent total gastrectomy or proximal gastrectomy at the Department of Gastrointestinal Surgery, First Affiliated Hospital of Dalian Medical University from January 2020 to November 2022. A total of 57 cases were initially included, but based on the subsequent inclusion and exclusion criteria, 52 cases were ultimately included.

### Inclusion criteria

*Age 18 years or older;*

*Preoperative pathological diagnosis of gastric cancer and undergoing curative total gastrectomy or proximal gastrectomy;*

*No history of other malignant tumors.*

### Exclusion criteria

*Incomplete clinical or pathological data;*

*Presence of incurable factors, such as liver metastases or peritoneal metastases;*

*Receipt of neoadjuvant therapy prior to surgery;*

*Presence of severe comorbidities in cardiovascular, respiratory, or neurological systems;*

*Refusal to participate or failure to provide informed consent.*

### Data collected

Preoperatively, basic patient information including age and gender was collected. Intraoperatively, the presence of the PGA was assessed, and if identified, measurements were taken for the length of the PGA and the distance from its root to the celiac trunk. Additionally, lymph nodes around the PGA were dissected. Postoperatively, data regarding tumor location, longitudinal tumor diameter, macroscopic and histological types, presence of vascular and nerve infiltration, T-stage, N-stage, total number of dissected lymph nodes, total number of metastatic lymph nodes, and metastasis status of other regional lymph nodes were obtained from the resected specimens and pathological reports of the patients.

### Classification criteria for partial data

Age: <70 years and ≥ 70 years. Tumor location: The stomach is anatomically divided into three parts: upper portion, middle portion, and lower portion. The division is determined by a line connecting the upper third of the greater and lesser gastric curvatures with another line connecting the lower third of the greater and lesser gastric curvatures. In cases where the center of the tumor is located within 2 cm above or below the esophagogastric junction, it is recorded as esophagogastric junction. Macroscopic type of the tumor: Type 0 represents early gastric cancer, characterized by cancer limited to the mucosa or submucosa. Advanced gastric cancer is classified into Borrmann types: Type I (Polypoid), Type II (Fungating, ulcerated with sharp raised margins), Type III (Ulcerated with poorly defined infiltrative margins), and Type IV (Infiltrative, predominantly intramural lesion, poorly demarcated). Pathological types: differentiated type mainly includes papillary adenocarcinoma, well-differentiated tubular adenocarcinoma, moderately differentiated tubular adenocarcinoma, etc.; undifferentiated type mainly includes poorly differentiated adenocarcinoma, signet ring cell carcinoma, mucinous adenocarcinoma, etc. T-stage and N-stage: according to the 8th edition of the AJCC/UICC TNM classification of gastric cancer [9], it is divided into T1, T2, T3, T4 and N1, N2, N3, respectively.

### Collection of essential data

The extent of lymphadenectomy performed during the operation in this study was either D1 + or D2 lymphadenectomy. The specific lymph node dissection varied depending on the stage of the disease and the extent of gastrectomy [10], but all patients underwent intraoperative dissection of the following regional lymph nodes: No.1, No.2, No.3, No.4sa, No.4sb, No.7, No.8a, No.9, and No.11.

Method for identifying the PGA during the operation: If there is a vascular branch originating from the splenic artery towards the upper posterior wall of the gastric body, it is considered as the PGA. In cases where the PGA is present, its anatomical features are measured, including the length of the PGA and the distance from the root of the PGA to the celiac trunk. These measurements were obtained through direct visual examination or laparoscopy using a tape measure.

**Fig. 1:**
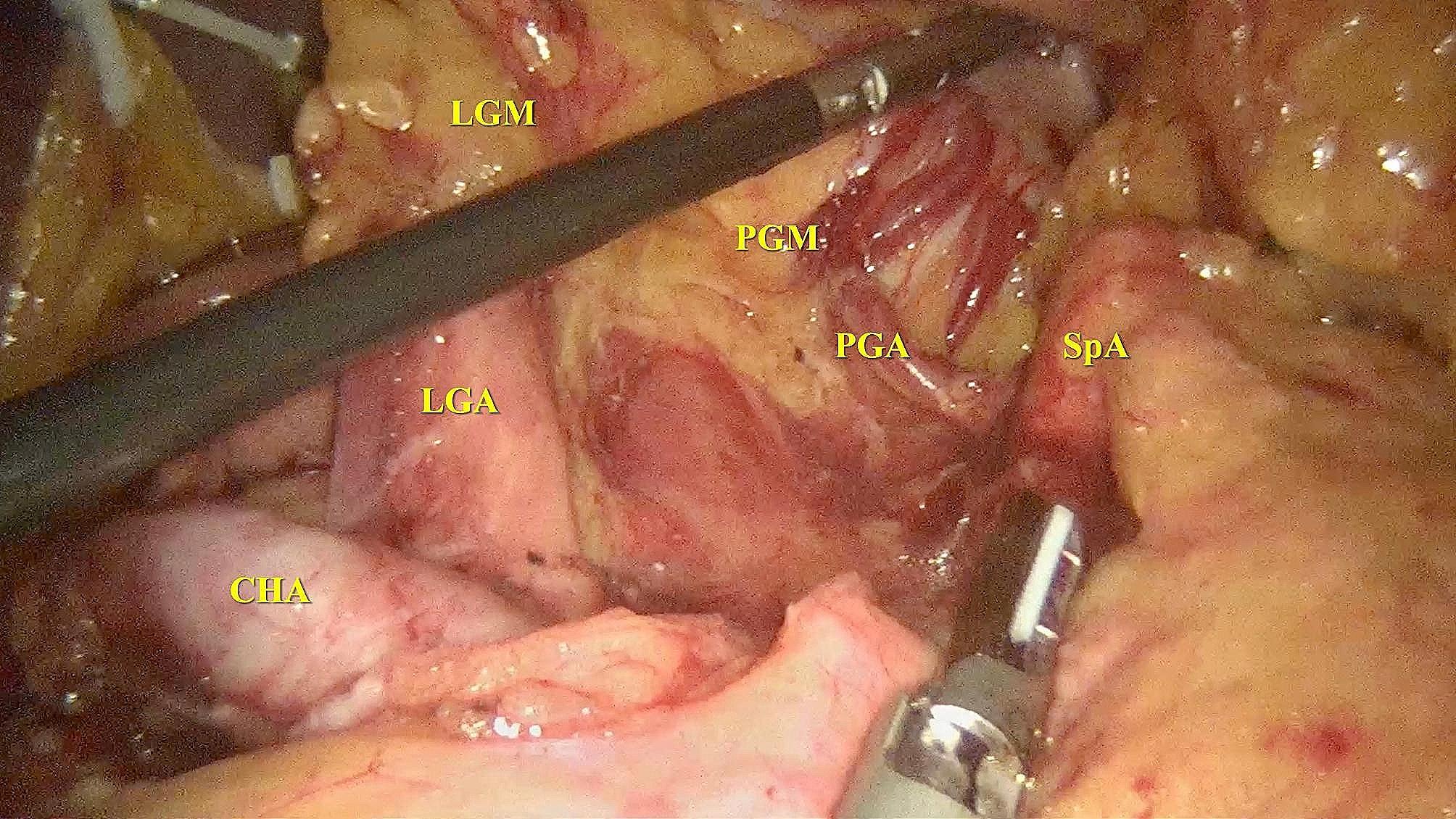
Posterior gastric mesentery and left gastric mesentery The picture shows the anatomical exposure of the posterior gastric mesentery and the left gastric mesentery when dissecting the lymph nodes located in the superior border of the pancreas during laparoscopic gastrectomy, showing that the PGA is enveloped by the posterior gastric mesentery. CHA: Common hepatic artery; LGA: Left gastric artery; LGM: Left gastric mesentery; PGA: Posterior gastric artery; PGM: Posterior gastric mesentery; SpA: Splenic artery

Method for obtaining lymph nodes around the PGA: The lymph nodes around the PGA are integral components of the posterior gastric mesentery. According to the membrane anatomy theory, the posterior gastric mesentery is one of the six mesenteries associated with the stomach. As shown in Fig. 1, it is situated in the upper region of the pancreas and envelops the posterior gastric vessels. It is adjacent to the short gastric mesentery on the left side and the left gastric mesentery on the right side, thereby anchoring the gastric fundus and body to the posterior abdominal wall of the upper pancreatic area [11]. The posterior gastric mesentery was entirely excised during the operation, and subsequently, the lymph nodes around the PGA were meticulously isolated from other regional lymph nodes during the postoperative handling of the gross specimens. These lymph nodes were then sent separately for pathological examination. It is crucial to differentiate between the lymph nodes around the PGA and the No.11 lymph nodes during the separation process. The No.11 lymph nodes correspond to the tissues encompassing the splenic artery, while the lymph nodes around the PGA pertain to the tissues situated between the origin of the PGA and the point at which it bifurcates into the posterior wall of the stomach.

### Statistical analysis

IBM SPSS Statistics 26 was used as the statistical analysis software in this study. Normality tests were performed on the quantitative data. Normally distributed quantitative data were presented as mean ± standard deviation [$\overline{X}\pm S$] and compared using t-tests. Non-normally distributed quantitative data were presented as median (lower quartile, upper quartile) [*M* (*P*_25_, *P*_75_)] or median (minimum to maximum) [*M* (*X*_min_ ∼ *X*_max_)] and compared using the Wilcoxon signed rank sum test. Qualitative data were presented using percentages and frequencies and compared using the chi-squared test. Univariate analysis employed the appropriate test method corresponding to the type of data. Multivariate analysis was conducted using either the forward-LR or backward-LR method of binary logistic regression analysis. A significance level of *P*<0.05 was used to determine the statistical significance of the observed differences in this study.

## Result

### General information

This study included a total of 52 cases, consisting of 38 males and 14 females. The median age was 65 years (range: 34–83). Among the 52 patients, 39 (75%) were found to have the PGA during the operation and underwent dissection of the lymph nodes around the PGA. Post-operative pathological examination revealed the presence of 36 lymph nodes around the PGA in the examined tissues from 20 patients with PGA. Among them, 2 patients had 3 metastatic lymph nodes around the PGA.

### Anatomical features and existence of the PGA

The PGA was detected during surgery in 39 out of 52 patients (75.0%) with gastric cancer. The PGA had a mean length of 3.5 ± 0.8 cm and a mean distance from the root of the PGA to the celiac trunk of 6.7 ± 1.7 cm. Table 1 presents the anatomical features of the PGA in 39 patients with gastric cancer, categorized by gender. No statistically significant differences were observed (all *P* values > 0.05).

**Table 1:** Anatomical features of the PGA

	*n*	Length of the PGA [cm, $\overline{X}\pm S$]	Statistical quantity	Distance from the root of the PGA to the celiac trunk [cm, $\overline{X}\pm S$]	Statistical quantity
Total	39	3.5$\pm$0.8		6.7$\pm$1.7	
Gender			*t* = 0.9 *P* = 0.371		*t* = 0.7 *P* = 0.515
Male	27	3.6$\pm$0.9		6.9$\pm$1.9	
Female	12	3.3$\pm$0.7		6.5$\pm$1.2	

*P*-value < 0.05 is considered as significant difference

Abbreviations: *PGA* Posterior gastric artery

The presence or absence of PGA in 52 patients with gastric cancer enrolled in this study was analyzed on a univariate basis with respect to various clinicopathological factors (For complete results of statistical analyses, please see Supplementary Tables 1 and Supplementary Table 2, Additional File 1). The results indicated no significant association between the presence of PGA and any clinicopathological factors, including gender, age, tumor location, macroscopic type, pathological type, presence of vascular infiltration, presence of nerve infiltration, T-stage, N-stage, longitudinal tumor diameter, total number of dissected lymph nodes, and total number of metastatic lymph nodes (all *P* values > 0.05).

Our study also performed the statistical analysis examining the relationship between each regional lymph node metastasis and the presence of the PGA (For complete results of statistical analyses, please see Supplementary Table 3, Additional File 1). The findings demonstrated no significant association between the presence of the PGA and any regional lymph node metastasis (all *P* values > 0.05).

### Analysis of factors for detection of lymph nodes around the posterior gastric artery

Post-operative pathological examination revealed the presence of 36 lymph nodes around the PGA in 20 out of 39 patients (51.3%) who underwent dissection of lymph nodes around the PGA. Tables 2 and 3 present the results of univariate analysis conducted to examine the detection of lymph nodes around the PGA in 39 patients with the PGA. The macroscopic type of the tumor showed a significant association with the detection of lymph nodes around the PGA (*P* = 0.007). Patients with macroscopic type of Borrmann type III and IV had a significantly higher proportion of detected lymph nodes around the PGA compared to patients with type of early gastric cancer and Borrmann type I and II. Moreover, the total number of dissected lymph nodes showed a significant association with the detection of lymph nodes around the PGA (*P* = 0.022). The group with detected lymph nodes around the PGA had a larger median total number of dissected lymph nodes.

**Table 2:** Univariate analysis of the detection of lymph nodes around the PGA in 39 patients with the PGA

Clinicopathological factors	*n*	Detected lymph nodes around the PGA [n (%)]	*P*-value (Fisher’s exact test)
Gender			1.000
Male	27	14 (51.9)	
Female	12	6 (50.0)	
Age			0.320
< 70	26	15 (51.7)	
≥ 70	13	5 (38.5)	
Tumor location			0.122
Esophagogastric junction	9	2 (22.2)	
Upper portion	16	9 (56.3)	
Middle portion	9	7 (77.8)	
Lower portion	5	2 (40.0)	
Macroscopic type			0.007
Type 0	10	2 (20.0)	
Borrmann type I and II	5	1 (20.0)	
Borrmann type III and IV	24	17 (70.8)	
Pathological type			1.000
Differentiated type	22	11 (50.0)	
Undifferentiated type	17	9 (52.9)	
Vascular infiltration			0.748
No	24	13 (54.2)	
Yes	15	7 (46.7)	
Nerve infiltration			0.752
No	19	9 (47.4)	
Yes	20	11 (55.0)	
T-stage			0.128
T1	10	2 (20.0)	
T2	3	2 (66.7)	
T3	17	11 (64.7)	
T4	9	5 (55.6)	
N-stage			0.374
N0	20	8 (40.0)	
N1	6	4 (66.7)	
N2	5	4 (80.0)	
N3	8	4 (50.0)	

*P*-value < 0.05 is considered as significant difference

Abbreviations: *PGA* Posterior gastric artery

**Table 3:** Univariate analysis of the detection of lymph nodes around the PGA in 39 patients with the PGA

Clinicopathological factors	Detected lymph nodes around the PGA	Did not detect lymph nodes around the PGA	Z or t	*P*-value
Longitudinal tumor diameter [cm, $\overline{X}\pm S$]	5.3$\pm$2.7	3.9$\pm$2.5	*t*=-1.6	0.109
Total number of dissected lymph nodes [n, *M* (*P*_25_, *P*_75_)]	30.5 (26.3, 43.5)	26.0 (13.0, 31.0)	*Z*=-2.3	0.022
Total number of metastatic lymph nodes [n, *M* (*P*_25_, *P*_75_)]	1.0 (0.0, 5.8)	0.0 (0.0, 3.0)	*Z*=-1.1	0.290
Length of the PGA [cm, $\overline{X}\pm S$]	3.5$\pm$0.9	3.5$\pm$0.8	*t* = 0.1	0.922
Distance from the root of the PGA to the celiac trunk [cm, *M* (*P*_25_, *P*_75_)]	7.0 (6.0, 7.9)	7.0 (5.5, 7.5)	*Z*=-0.3	0.754

*P*-value < 0.05 is considered as significant difference

Abbreviations: *PGA* Posterior gastric artery

Our study also performed a multivariate binary logistic regression analysis using the forward-LR method, where the detection or non-detection of lymph nodes around the PGA is the dependent variable, and each clinicopathological factor is considered as an independent variable. The multivariate analysis revealed that a higher number of total dissected lymph nodes was an independent relevant factor associated with the detection of lymph nodes around the PGA (*OR* = 1.105, *95%CI*: 1.019–1.199, *P* = 0.016). With each one increase in the total number of dissected lymph nodes, there is a 10.5% increase in the likelihood of detecting lymph nodes around the PGA.

### Analysis of risk factors for metastasis of the lymph nodes around the PGA

Among the 39 patients who underwent dissection of lymph nodes around the PGA, metastasis of the lymph nodes around the PGA was detected in 2 patients. Table 4 presents selected clinicopathological data of two patients who had metastasis of the lymph nodes around the PGA.

**Table 4:** Clinicopathological data of the patients with metastasis of the lymph nodes around the PGA

**Case**	**Gender**	**Age**	**Tumor location**	**Longitudinal tumor diameter**	**Macroscopic type**	**Histologic type**
1	Male	67	Esophagogastric junction	6 cm	Borrmann III	Signet-ring cell carcinoma
2	Male	70	Upper portion	12 cm	Borrmann IV	Poorly differentiated adenocarcinoma
**Case**	**pTNM**		**Total number of metastatic lymph nodes/Total number of dissected lymph nodes**	**Number of metastatic lymph nodes around the PGA /Number of dissected lymph nodes around the PGA**	**Other metastatic regional lymph nodes**	
1	pT4aN3bM0		16/30	2/2	No.1、No.3、No.7、No.8a、No.9、No.11、No.20	
2	pT4aN2M0		6/63	1/2	No.3、No.11	

*P*-value < 0.05 is considered as significant difference

Abbreviations: *PGA* Posterior gastric artery

Table 5 present part of the results of univariate analysis on metastasis of the lymph nodes around the PGA in 39 patients with the PGA (For other part of statistical analyses, please see Supplementary Table 4, Additional File 1). The findings revealed a significant association between the total number of metastatic lymph nodes and metastasis of the lymph nodes around the PGA (*P* = 0.043). The group of lymph nodes around the PGA with metastasis had a higher median total number of metastatic lymph nodes.

**Table 5:** Univariate analysis of the metastasis of the lymph nodes around the PGA in 39 patients with the PGA

Clinicopathological factors	Lymph nodes around the PGA with metastasis	Lymph nodes around the PGA without metastasis	Z	*P*-value
Longitudinal tumor diameter [cm, *M* (*X*_min_ ∼ *X*_max_)]	9.0 (6.0 ∼ 12.0)	3.7 (0.6 ∼ 10.0)	-1.7	0.085
Total number of metastatic lymph nodes [n, *M* (*X*_min_ ∼ *X*_max_)]	11.0 (6.0 ∼ 16.0)	0.0 (0.0 ∼ 16.0)	-2.0	0.043
Length of the PGA [cm, *M* (*X*_min_ ∼ *X*_max_)]	3.0 (3.0 ∼ 4.0)	3.5 (2.0 ∼ 6.0)	-1.0	0.311
Distance from the root of the PGA to the celiac trunk [cm, *M* (*X*_min_ ∼ *X*_max_)]	6.5 (7.0 ∼ 7.0)	7.0 (2.0 ∼ 11.0)	-0.8	0.438

*P*-value < 0.05 is considered as significant difference

Abbreviations: *PGA* Posterior gastric artery

Table 6 presents the statistical analysis of each regional lymph node metastasis and metastasis of the lymph nodes around the PGA. The results indicated a significant association between No.3 and No.11 lymph nodes metastases and metastasis of the lymph nodes around the PGA (*P* = 0.028, *P* = 0.020, respectively).

**Table 6:** Regional lymph node metastasis status of 39 patients with or without the metastasis of the lymph nodes around the PGA

Regional lymph node	*n*	Lymph nodes around the PGA with metastasis [n (%)]	*P*-value (Fisher’s exact test)
No.1			0.331
Negative	32	1 (3.1)	
Positive	7	1 (14.3)	
No.2			1.000
Negative	36	2 (5.6)	
Positive	3	0 (0.0)	
No.3			0.028
Negative	32	0 (0.0)	
Positive	7	2 (28.6)	
No.4sa			1.000
Negative	37	2 (5.4)	
Positive	2	0 (0.0)	
No.4sb			1.000
Negative	38	2 (5.3)	
Positive	1	0 (0.0)	
No.7			0.413
Negative	30	1 (3.3)	
Positive	9	1 (11.1)	
No.8a			0.197
Negative	35	1 (2.9)	
Positive	4	1 (25.0)	
No.9			0.101
Negative	37	1 (2.7)	
Positive	2	1 (50.0)	
No.11			0.020
Negative	33	0 (0.0)	
Positive	6	2 (33.3)	

*P*-value < 0.05 is considered as significant difference

Abbreviations: *PGA* Posterior gastric artery

A multivariate binary logistic regression analysis was conducted to determine the association between metastasis of the lymph nodes around the PGA and clinicopathological factors as well as each regional lymph node metastasis. However, no significant independent risk factor for metastasis of the lymph nodes around the PGA was identified in the results.

## Discussion

Anatomical observations reveal that the splenic artery, originating from the celiac trunk, typically gives rise to downward pancreatic branches without any upward branches throughout its course. However, occasional upward-running branches [5], such as the PGA, can be observed. The incidence, origin, course, and distribution of the PGA vary across studies due to differences in its definition and research methods [6, 7, 12, 13]. According to the prevailing view, the PGA usually originates from the splenic artery, posterior to the gastric body, and ascends behind the peritoneum of the lesser sac towards the gastric fundus, reaching the posterior surface of the upper gastric body. In this study, the PGA is defined following this perspective, referring to the vessels originating from the splenic artery and distributed in the upper posterior wall of the gastric body. Unlike most prior studies that relied on gross autopsy, the observations of the PGA in this study were obtained from living patients during surgical operations for gastric cancer treatment, ensuring no additional harm to the patients.

The stomach possesses a highly abundant blood supply, with the PGA playing a primary role in supplying blood to the upper portion. The left gastric artery is the most significant nutrient vessel in the upper portion of the stomach, followed by the PGA, the short gastric artery, and the left gastro-omental artery [12]. In this study, the PGA was found to have an incidence rate of 75.0%, with a mean length of 3.5 ± 0.8 cm and a mean distance from its root to the celiac trunk of 6.7 ± 1.7 cm. Due to the PGA’s high incidence and its intricate course, accurate identification during gastric surgery is crucial to avoid potential complications such as bleeding and tissue ischemia [13]. For instance, during a distal gastrectomy that involves the division of the left gastric artery and the left gastro-omental artery, the blood supply to the proximal remnant stomach primarily relies on the PGA and the short gastric artery. If the PGA is inadvertently severed, it can compromise the blood supply to the remnant stomach and impede anastomotic healing in the short term, even if collateral circulation gradually develops through the remaining vessels postoperatively [7].

Our study aimed to investigate the factors associated with the presence of the PGA by comparing the clinicopathological features, including the metastasis status of each regional lymph node, between the two groups: patients with and without the PGA among the 52 included patients with gastric cancer. The statistical analysis results did not reveal any statistically significant factors. However, the data analysis indicated a higher proportion of PGA presence in patients with No.11 lymph node metastasis compared to those without No.11 lymph node metastasis (85.7% vs. 73.3%, *P* = 0.815). Thus, the possibility of an association between No.11 lymph node metastasis and the presence of the PGA cannot be ruled out.

According to the 15th edition of the Japanese Classification of Gastric Carcinoma [8], well-defined regional lymph nodes surround all the major peri-gastric vessels except for the PGA. For example, the esophageal-cardiac branch of the left inferior phrenic artery is surrounded by No.2 lymph nodes, the short gastric artery is surrounded by No.4sa lymph nodes, the left gastro-omental artery is surrounded by No.4sb lymph nodes, the right gastric artery is surrounded by No.5 and No.3d lymph nodes, the right gastro-omental artery is surrounded by No.6 and No.4d lymph nodes, and the left gastric artery is surrounded by No.7, No.1, and No.3a lymph nodes. However, lymph nodes around the PGA are not currently defined. Due to this lack of clarity, during gastric cancer surgery, lymph nodes around the PGA may be dissected along with the nearby lymph nodes such as No.11 or No.2 lymph nodes, or they may not be dissected. Nonetheless, lymph nodes around the PGA may have clinical significance. Kato [14] has suggested the possibility of lymphatic metastases occurring along the PGA in gastric cancer, but it remains unclear whether lymph nodes exist around the PGA and whether the cancer can metastasize to these lymph nodes. Ishii et al. [6] conducted a study on PGA and its surrounding lymph nodes, confirming the presence of the PGA in 11 (52.4%) out of 21 autopsies. Among them, lymph nodes around the PGA were detected in 2 cases, resulting in a detection rate of 9.5%. This study represents the only known investigation that confirmed the presence of lymph nodes around the PGA during the time of our study.

Our study further elucidated the presence of lymph nodes around the PGA, building upon the findings of Ishii et al [6]. Through post-operative pathological examination, we detected 36 lymph nodes around the PGA in 20 (51.3%) out of the 39 patients who underwent dissection of lymph nodes around the PGA. Univariate analysis of detection of lymph nodes around the PGA revealed a significant association with the macroscopic tumor type and the total number of dissected lymph nodes (*P* = 0.007, *P* = 0.022, respectively). The proportion of detected lymph nodes around the PGA was significantly higher in patients with Borrmann type III and IV tumors (70.8%) compared to those with early gastric carcinoma and Borrmann type I and II tumors (20.0%). Borrmann type III and IV gastric cancers exhibit invasive growth patterns and distinct biological behavior, particularly Borrmann type IV tumors which tend to be more malignant [15]. Therefore, it is speculated that the difference in detecting lymph nodes around the PGA in patients with different macroscopic types may be related to these characteristics. Additionally, the median total number of dissected lymph nodes was 30.5 (26.3, 43.5) in the group with detected lymph nodes around the PGA, whereas it was 26.0 (13.0, 31.0) in the group without detected lymph nodes around the PGA (*P* = 0.022). Multifactorial analysis demonstrated that the total number of dissected lymph nodes was an independent relevant factor for the detection of lymph nodes around the PGA (*OR* = 1.105, *95% CI*: 1.019–1.199, *P* = 0.016), indicating that each one increase in the total number of dissected lymph nodes increased the likelihood of detecting lymph nodes around the PGA by 10.5%.

Our study demonstrated, for the first time, the occurrence of metastasis from gastric cancer to the lymph nodes around the PGA. Among the 39 patients who underwent dissection of lymph nodes around the PGA, 2 patients exhibited metastasis of these lymph nodes. Univariate analysis revealed that the median total number of metastatic lymph nodes in the group with metastasis of the lymph nodes around the PGA was 11.0 (6.0–16.0), which was higher than the 0.0 (0.0–16.0) observed in the group without metastasis of the lymph nodes around the PGA (*P* = 0.043). This finding suggests that an increase in the total number of metastatic lymph nodes in patients with gastric cancer elevates the risk of metastasis of the lymph nodes around the PGA. Additionally, there was a significant correlation between No. 3 and No. 11 lymph nodes metastases and metastasis of the lymph nodes around the PGA (*P* = 0.028 and *P* = 0.020, respectively). However, multivariate analysis did not identify independent risk factors for metastasis of the lymph nodes around the PGA. It is important to note that the analysis of risk factors was limited by the small number of cases with metastasis of the lymph nodes around the PGA and the overall sample size of only 39, which hindered the detection of additional factors with significant differences through statistical analysis. The data from the 2 patients with metastasis of the lymph nodes around the PGA revealed that both had a poorly differentiated pathological type and an advanced clinical stage.

The cumulative results of these statistical analyses suggest that the overall risk of metastasis of the lymph nodes around the PGA is low in patients with gastric cancer. However, this risk increases when there are metastases in No.3 and No.11 lymph nodes, advanced tumor stage, and extensive metastases in other regional lymph nodes. While our study provides the first confirmation of gastric cancer metastasis of the lymph nodes around the PGA, it is constrained by a small sample size, which limits the interpretability of the statistical analysis results.

This single-centre study has inherent limitations due to the small sample size and the potential presence of selection bias in case inclusion, thereby limiting its ability to fully represent the actual scenario. Future studies should incorporate a larger sample size for analysis and explore survival and prognosis in more depth.

## Conclusion

The PGA has a high incidence and requires careful identification and appropriate management to prevent vascular-related complications, including bleeding and tissue ischemia during surgeries involving the upper gastric region, pancreas, and spleen. In patients with gastric cancer, the overall risk of metastasis of the lymph nodes around the PGA is low. However, this risk increases with the presence of metastases in No.3 and No.11 lymph nodes, advanced tumor stage, and extensive metastases in other regional lymph nodes.

### Electronic supplementary material

Below is the link to the electronic supplementary material.

Supplementary Material 1

## Data Availability

No datasets were generated or analysed during the current study.
