# Supplementary material for: Clinical study of the posterior gastric artery and the lymph nodes around it in patients with gastric cancer
Source: World J Surg Oncol. 2024 Apr 10;22:90. doi: 10.1186/s12957-024-03373-x (PMC11005264; doi:10.1186/s12957-024-03373-x)
Supplement: Supplementary file 1 — Supplementary Material 1 [file 12957_2024_3373_MOESM1_ESM.docx]

*Supplementary Table 1 Clinicopathological features of 52 patients with or without the PGA*

| Clinicopathological factors | n | With the PGA  [n (%)] | 𝜒^2^ | *P*-value |
| --- | --- | --- | --- | --- |
|  |  |  |  |  |
| Gender |  |  | 0.521 | 0.470 |
| Male | 38 | 27 (71.1) |  |  |
| Female | 14 | 12 (85.7) |  |  |
| Age |  |  | 0.000 | 1.000 |
| < 70 | 34 | 26 (76.5) |  |  |
| ≥ 70 | 18 | 13 (72.2) |  |  |
| Tumor location |  |  | 3.866 | 0.261 |
| Esophagogastric junction | 11 | 9 (81.8) |  |  |
| Upper portion | 19 | 16 (84.2) |  |  |
| Middle portion | 16 | 9 (56.3) |  |  |
| Lower portion | 6 | 5 (83.3) |  |  |
| Macroscopic type |  |  | 2.620 | 0.234 |
| Type 0 | 16 | 10 (62.5) |  |  |
| Borrmann type I and Ⅱ | 5 | 5 (100.0) |  |  |
| Borrmann type Ⅲ and Ⅳ | 31 | 24 (77.4) |  |  |
| Pathological type |  |  | 2.564 | 0.109 |
| Differentiated type | 26 | 22 (84.6) |  |  |
| Undifferentiated type | 26 | 17 (65.4) |  |  |
| Vascular infiltration |  |  | 0.453 | 0.501 |
| No | 34 | 24 (70.6) |  |  |
| Yes | 18 | 15 (83.3) |  |  |
| Nerve infiltration |  |  | 0.642 | 0.423 |
| No | 27 | 19 (70.4) |  |  |
| Yes | 25 | 20 (80.0) |  |  |
| T-stage |  |  | 2.105 | 0.580 |
| T1 | 16 | 10 (62.5) |  |  |
| T2 | 3 | 3 (100.0) |  |  |
| T3 | 22 | 17 (77.3) |  |  |
| T4 | 11 | 9 (81.8) |  |  |
| N-stage |  |  | 0.759 | 0.961 |
| N0 | 28 | 20 (71.4) |  |  |
| N1 | 7 | 6 (85.7) |  |  |
| N2 | 6 | 5 (83.3) |  |  |
| N3 | 11 | 8 (72.7) |  |  |

P-value < 0.05 is considered as significant difference.

Abbreviations: *PGA* Posterior gastric artery.

*Supplementary Table 2 Clinicopathological features of 52 patients with or without the PGA*

| Clinicopathological factors | With the PGA | Without the PGA | *Z* | *P*-value |
| --- | --- | --- | --- | --- |
|  |  |  |  |  |
| Longitudinal tumor diameter [cm, *M* (*P*_25_, *P*_75_)] | 4.0 (2.5, 6.0) | 3.5 (1.3, 5.2) | -1.059 | 0.289 |
| Total number of dissected lymph nodes [n, *M* (*P*_25_, *P*_75_)] | 30.0 (23.0, 35.0) | 32.0 (20.5, 39.0) | -0.635 | 0.526 |
| Total number of metastatic lymph nodes [n, *M* (*P*_25_, *P*_75_)] | 0.0 (0.0, 5.0) | 0.0 (0.0, 9.0) | -0.161 | 0.872 |

P-value < 0.05 is considered as significant difference.

Abbreviations: *PGA* Posterior gastric artery.

*Supplementary Table 3 Regional lymph node metastasis status of 52 patients with or without the PGA*

| Regional lymph node | n | With the PGA  [n (%)] | *P*-value  (Fisher’s exact test) |
| --- | --- | --- | --- |
|  |  |  |  |
| No.1 |  |  | 0.697 |
| Negative | 42 | 32 (76.2) |  |
| Positive | 10 | 7 (70.0) |  |
| No.2 |  |  | 0.589 |
| Negative | 47 | 36 (76.6) |  |
| Positive | 5 | 3 (60.0) |  |
| No.3 |  |  | 0.147 |
| Negative | 40 | 32 (80.0) |  |
| Positive | 12 | 7 (58.3) |  |
| No.4sa |  |  | 0.257 |
| Negative | 48 | 37 (77.1) |  |
| Positive | 4 | 2 (50.0) |  |
| No.4sb |  |  | 0.151 |
| Negative | 49 | 38 (77.6) |  |
| Positive | 3 | 1 (33.3) |  |
| No.7 |  |  | 1.000 |
| Negative | 40 | 30 (75.0) |  |
| Positive | 12 | 9 (75.0) |  |
| No.8a |  |  | 0.632 |
| Negative | 46 | 35 (76.1) |  |
| Positive | 6 | 4 (66.7) |  |
| No.9 |  |  | 0.257 |
| Negative | 48 | 37 (77.1) |  |
| Positive | 4 | 2 (50.0) |  |
| No.11 |  |  | 0.664 |
| Negative | 45 | 33 (73.3) |  |
| Positive | 7 | 6 (85.7) |  |

P-value < 0.05 is considered as significant difference.

Abbreviations: *PGA* Posterior gastric artery.

*Supplementary Table 4 Univariate analysis of the metastasis to the lymph nodes around the PGA in 39 patients with the PGA*

| Clinicopathological factors | n | Lymph nodes around the PGA with metastasis  [n (%)] | *P*-value  (Fisher’s exact test) |
| --- | --- | --- | --- |
|  |  |  |  |
| Gender |  |  | 1.000 |
| Male | 27 | 2 (7.4) |  |
| Female | 12 | 0 (0.0) |  |
| Age |  |  | 1.000 |
| < 70 | 26 | 1 (3.8) |  |
| ≥ 70 | 13 | 1 (7.7) |  |
| Tumor location |  |  | 1.000 |
| Esophagogastric junction | 9 | 1 (11.1) |  |
| Upper portion | 16 | 1 (6.3) |  |
| Middle portion | 9 | 0 (0.0) |  |
| Lower portion | 5 | 0 (0.0) |  |
| Macroscopic type |  |  | 1.000 |
| Type 0 | 10 | 0 (0.0) |  |
| Borrmann type I and Ⅱ | 5 | 0 (0.0) |  |
| Borrmann type Ⅲ and Ⅳ | 24 | 2 (8.3) |  |
| Pathological type |  |  | 0.184 |
| Differentiated type | 22 | 0 (0.0) |  |
| Undifferentiated type | 17 | 2 (11.8) |  |
| Vascular infiltration |  |  | 1.000 |
| No | 24 | 1 (4.2) |  |
| Yes | 15 | 1 (6.7) |  |
| Nerve infiltration |  |  | 0.487 |
| No | 19 | 0 (0.0) |  |
| Yes | 20 | 2 (10.0) |  |
| T-stage |  |  | 0.130 |
| T1 | 10 | 0 (0.0) |  |
| T2 | 3 | 0 (0.0) |  |
| T3 | 17 | 0 (0.0) |  |
| T4 | 9 | 2 (22.2) |  |
| N-stage |  |  | 0.166 |
| N0 | 20 | 0 (0.0) |  |
| N1 | 6 | 0 (0.0) |  |
| N2 | 5 | 1 (20.0) |  |
| N3 | 8 | 1 (12.5) |  |

P-value < 0.05 is considered as significant difference.

Abbreviations: *PGA* Posterior gastric artery.
